# Subjective health status of multimorbidity: verifying the mediating effects of medical and assistive devices

**DOI:** 10.1186/s12939-018-0880-3

**Published:** 2018-11-12

**Authors:** Munjae Lee, Kichan Yoon, Kyu-Sung Lee

**Affiliations:** 1 Department of Medical Device Management and Research, SAIHST, Sungkyunkwan University, Seoul, Korea; 2 Research Center, Social Security Information Institute, Social Security Information Service, Seoul, Korea; 3 Department of Urology, Samsung Medical Center, Sungkyunkwan University School of Medicine, Seoul, Korea

**Keywords:** Subjective health status, Multimorbidity, Mediating effect, Medical devices, Assistive devices

## Abstract

**Background:**

This study aimed to verify the mediating effect of using assistive devices as a factor that alleviates the relationship between multimorbidity and subjective health status.

**Methods:**

This study used three-year data (2011–2013) from the Korea Health Panel (KHP). The data were jointly collected by the consortium of the National Health Insurance Service and Korea Institute for Health and Social Affairs.

**Results:**

The mediating effect of using assistive devices was verified, but the direction of the effect was deteriorated subjective health. In other words, in terms of the impact of multimorbidity on subjective health, using assistive devices had a negative impact (−) on subjective health.

**Conclusions:**

The current assessment system for medical devices, narrow scope for choice of assistive devices, and limited scope of health insurance benefits must change to ultimately lead to a positive mediating effect on using medical devices and on subjective health satisfaction of patients with chronic diseases. A system that embraces all ages and generations must be developed. To this end, it is necessary to expand the scope of medical devices and insurance payment in long-term care insurance for elderly users, as well as the active meaning of medical devices in terms of health insurance.

## Introduction

The national demand for medical devices is expected to rise rapidly with the aging population and growing needs for well-being. Developing and supplying medical devices with high added value and ripple effect are necessary to meet new demands [1]. Specifically, assistive devices mostly help patients with chronic and degenerative diseases, such as rheumatoid arthritis, to live through their daily lives in an aging society. Moreover, there is a greater need for assistive devices if these patients have multiple diseases [2–5].

For example, the United States, which is an aging society, has increased its supply of assistive devices to guarantee the daily life activities of the elderly made functionally imperfect by chronic diseases [6, 7]. Several studies overseas have examined the ways to improve quality of life and daily activities through assistive devices [8, 9]. Hartke, Prohaska [10] demonstrated that people with poor health conditions due to an illness tend to use assistive devices and multiple medical appliances.

In using assistive devices, people with a disease or disability can lead an independent life [11, 12]. Assistive devices may also improve users’ rehabilitation and social participation, leading to satisfaction with daily life [13, 14] and an enhanced quality of life [15]. However, those who use assistive devices may also experience more pain and a lower level of subjective satisfaction compared with those who do not [16]. Particularly, first-time users may be unsatisfied with changes in their social relationships attributable to emotional reasons [17]. Therefore, assistive device users may simultaneously experience conflicting feelings of opportunities and limitations as well as security and worry, which may result in both positive and negative effects on their subjective health satisfaction [18].

Subjective health satisfaction is a concept that embraces emotional and social satisfaction, including not only physical health from using assistive devices but also improved quality of life and satisfaction with daily life. Therefore, using assistive devices, including medical appliance, may affect subjective health status [19, 20]. Many studies have used subjective health as a suitable index to measure individual health, such as predicted mortality [21, 22]. Indeed, it is an indicator that can be used as a health outcome in studies on health and medical services, with proven validity [23, 24]. However, the subjective and objective health status of assistive device users may not necessarily be similar. Depending on the socio-cultural norms of a country, the use of assistive devices can have a labeling effect; therefore, users may not be personally and socially accepted, and the physical convenience provided by assistive devices may be counteracted [17].

Meanwhile, chronic disease is known as a factor that intensifies the burden of disease for the population in an aging society and directly leads to death [25]. Once it has occurred, a chronic disease is impossible to cure completely and requires constant care to delay additional illness [26]. Moreover, when it leads to two or more conditions of multimorbidity, it rapidly aggravates an individual’s health [27–30]. Along with aging, multimorbidity is a global trend [31], requiring multilateral approaches to prevention and care in terms of health and medical services [32]. Multimorbidity constantly aggravates individual health conditions and is rising as a factor that shortens life expectancy in an aging society [33]. There have been various studies on chronic disease and subjective health [34, 35]. Multimorbidity is a factor with a negative impact on individual health as well as death. There is an increasing need for an intermediate mechanism that delays additional multimorbidity and reduces subjective health deterioration, in case there is development of a chronic disease.

At present, the mediating effect of assistive devices in terms of causal relations between chronic disease and satisfaction with subjective health has not been verified. The concept of assistive devices is not clearly developed, but in Korea, the relevant standard has been established according to the application of the Act on Long-term Care Insurance for Senior Citizens. Assistive devices are defined as devices to help daily life and physical activities of the beneficiaries of long-term care insurance, either lent directly to the ones in need or provided in the form of services, such as rehabilitation, with the provider visiting their homes with the devices. There are nine types of assistive devices for purchase, such as portable toilets, bath chairs, and adult walkers, and eight types of assistive devices for rental, such as manual wheelchairs, adjustable beds, and portable bathtubs [36].

Studies on assistive devices in Korea have focused on satisfaction, demand, and effects of use [37]. Research that examined satisfaction with using assistive devices categorized use into activities of daily living (ADL) and instrumental activities of daily living (IADL), and emphasized the need for assistive devices in daily living while also verifying the preference order for assistive devices in each category [38, 39]. Kang [40] reported that using such devices involves a negative mindset, such as social stigma and embarrassment, although practical use results in a positive mindset that makes the daily living of elderly users more functionally convenient. In developed countries with an aging population, assistive devices are found effective in promoting the quality of life and social functions of people with hearing impairment [41, 42]. Kim, Nam [43] classified satisfaction with using assistive devices into convenience, safety and solidity, functionality and effectiveness, and cost relevance; they revealed that although there are differences depending on the type of assistive device, the satisfaction decreases owing to the burden of use. The increase in aging-related chronic disease will lead to the increased use of assistive devices, which may have psychologically negative effects but may also improve satisfaction with subjective health in daily life [44].

In sum, use of assistive devices has a positive effect on the quality of the user’s life and daily living. However, many previous studies have been focused on the impact of multimorbidity on subjective health status, as well as on satisfaction, the need for and the improvement of assistive devices. In other words, there is insufficient research on the effect of assistive devices on the individual’s disease status. Therefore, this study aimed to verify the mediating effect of using assistive devices as a factor that alleviates the relationship between multimorbidity and subjective health status.

## Methods

### Data source and research participants

This study used three years of data, from 2011 to 2013, from the Korea Health Panel (KHP) jointly collected by the consortium of the National Health Insurance Service and Korea Institute for Health and Social Affairs. The target group comprised the 47,746 participants of the KHP study, among whom 13,189 (27.6%) participants from the three abovementioned years were selected, excluding missing values and outliers. To rule out missing values, the method of deleting all values measured where missing values occurred (listwise deletion) was used, as the panel using assistive devices is relatively restricted.

### Hypothesis

**Fig. 1:**
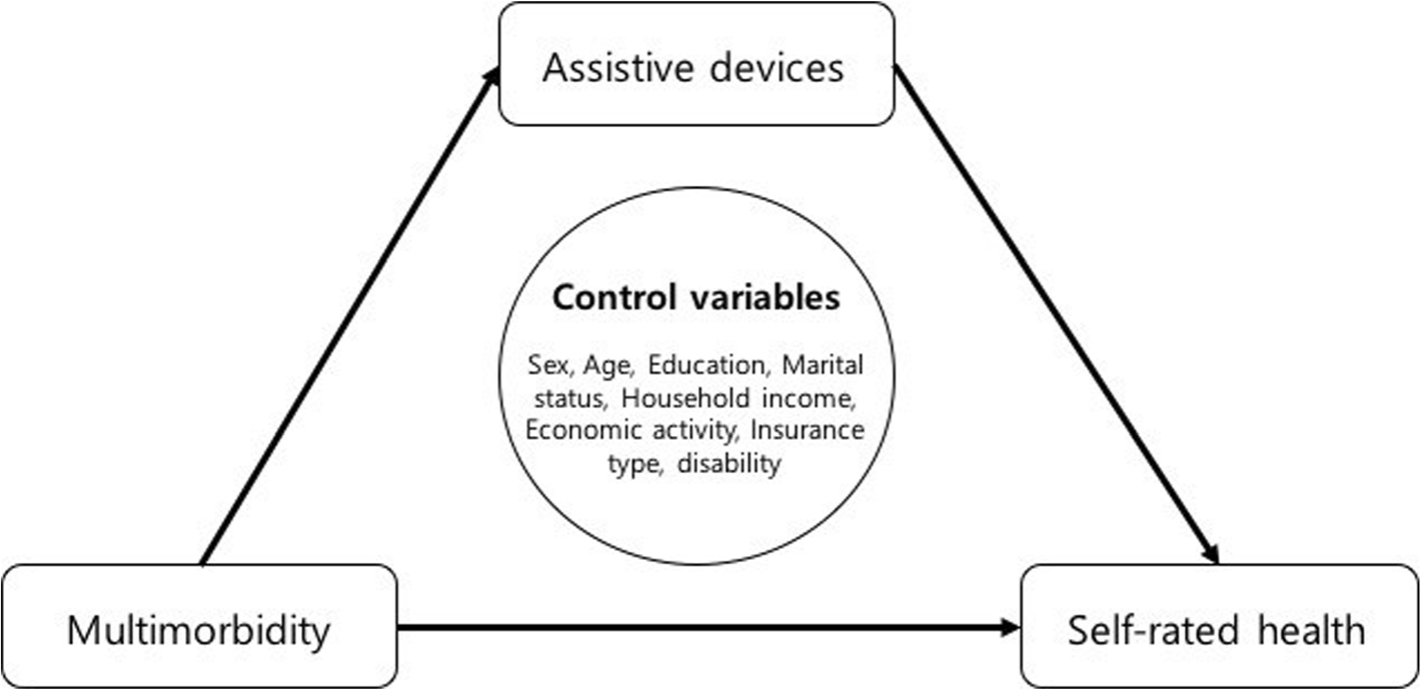
Research model for association between multimorbidity and self-rated health mediated by assistive devices

Multimorbidity was selected as the independent variable and subjective health status as the dependent variable. Use of medical and assistive devices was used as the mediating variable for multimorbidity and subjective health status. The specific analytical model used in the study is presented in Fig. 1.

The following hypotheses were proposed to analyze the impact of multimorbidity on subjective health status and the mediating effect of using medical and assistive devices.

Hypothesis 1: Multimorbidity will have a negative impact on subjective health status.

Hypothesis 2: Multimorbidity will have an impact on using medical and assistive devices.

Hypothesis 3: Using medical and assistive devices will have an impact on subjective health status.

Hypothesis 4: Using medical and assistive devices will determine the mediating effects between multimorbidity and subjective health status.

### Description of variables

The variables used in this study were described as follows (Table 1). Multimorbidity, the independent variable, was classified into the population without any chronic disease and that with one, two, and three or more chronic diseases. Although multimorbidity refers to two or more diseases, the group with three or more chronic diseases was separated to clarify the distinction in terms of severity of multimorbidity, given that a person with two or more chronic diseases will have drastically deteriorating health [27]. In this study, multimorbidity was based on the panel of three or more chronic diseases requiring assistive devices rather than on the severity of a patient’s health condition.

**Table 1:** Description of variables

Type	Variables	
Dependent variable	Subjective health status	1: very good
		2: good
		3: fair
		4: poor
		5: very poor
Independent variable	Multimorbidity	0, 1, 2, 3+
Mediator	Use of medical device	0: no use
		1: use
Control variables	Sex	0: female
		1: male
	Age	Continuous variable
	Marital status	0: single
		1: married
		2: etc. (divorced, widowed)
	Education	0: under elementary
		1: under high school
		2: over university
	Household income	1: quantile 1 (low income)
		2: quantile 2
		3: quantile 3
		4: quantile 4
		5: quantile 5 (high income)
	Economic activity	0: no
		1: yes
	Type of insurance	0: assistance
		1: national health insurance
	Disability	0: no
		1: yes

Subjective health status, the dependent variable, was based on the data from one survey item in the KHP. Subjective health status was rated on a five-point scale, with 1 point indicating “very good” and 5 points “very poor.”

Medical and assistive devices, the mediator variable of this study, used variables related to medical devices in the KHP data. Medical devices indicated assistive devices, such as wheelchairs and walking sticks; non-specialized medical devices, such as wheelchairs, walking sticks, and hospital mattress; and undefined specialty medical devices, such as MRI and CT. They were referred to as medical devices to promote the understanding of the participants in the survey [45]. The relevant variables were indicated by dividing the participants into use and non-use groups.

Socio-demographic variables, such as sex, age, marital status, education level, and household income level, insurance type, economic activity, and disability status, were used for control variables.

### Statistical analysis

Statistical analysis was conducted after data extraction and coding with STATA ver. 12 SE. Data analysis was performed in the following order. First, descriptive statistical analysis was conducted to identify the demographic characteristics of the participants. Second, the between-group differences of the independent, dependent, and mediator variables were determined according to the demographic characteristics. Third, logit regression analysis was performed to identify the relationship between multimorbidity and experiences of using medical and assistive devices, and panel regression analysis, to identify the relationship between multimorbidity and subjective health satisfaction as well as between experiences of using medical and assistive devices and their impact on subjective health satisfaction. Moreover, the mediating effect on the experiences of using medical and assistive devices was verified using Sobel test, according to the method by Baron and Kenny based on multiple regression analysis [46].

Through panel regression analysis, the three-year variants of independent, mediating, and dependent variables were converted, and the fixed effect model was used to identify the impact of such time-variant chronic diseases and experience of using assistive devices on subjective health satisfaction. The fixed effect model was appropriately used, as the panel data in this study were balanced with even intervals between survey timings, identical participants, and survey tools [47]. Meanwhile, a logit regression analysis was conducted using the panel data to verify the impact of chronic diseases on the experience of using medical devices.

## Results

### General characteristics

The general characteristics of the research participants are listed in Table 2. First, the portion of patients with multimorbidity gradually increased. Moreover, the subjective health status score increased from 2.64 in 2011 to 2.69 in 2013, moving further toward unhealthiness. Individuals with experience in using medical and assistive devices accounted for 4.59, 4.27, and 6.12% of the sample each year, respectively. There were more women than men, and the average age was 52.54 years. Most of the participants were married (71.21%), followed by single, and others (divorced or bereaved). For education level, most of them were high school graduates (48.09%), followed by college graduates or higher (29.21%), and elementary school graduates or lower (22.70%).For household income, the data were divided by income quantile for all participants of the KHP, and thus showed a relatively even distribution. Further, most participants engaged in economic activities (60.11%), and most of them were also users of the National Health Insurance (95.56%). Finally, around 6% of the participants had disabilities.

**Table 2:** General characteristics of research participants, Unit: n (%)

Variables		Year		
		2011	2012	2013
MCD	0	4479 (38.10)	4101 (37.22)	3627 (34.42)
	1	2310 (19.65)	2170 (19.70)	2058 (19.53)
	2	1638 (13.93)	1470 (13.34)	1414 (13.42)
	3+	3328 (28.31)	3277 (29.74)	3437 (32.62)
SHS	Continuous	2.64 (0.86)	2.67 (0.86)	2.69 (0.82)
MD	No	11,216 (95.41)	10,547 (95.73)	9891 (93.88)
	Yes	539 (4.59)	471 (4.27)	645 (6.12)
Sex	Female	6447 (54.84)	6042 (54.84)	5786 (54.92)
	Male	5308 (45.16)	4976 (45.16)	4750 (45.08)
Age		51.03 (16.49)	51.91 (16.67)	52.54 (16.98)
Marital status	Single	1892 (16.10)	1764 (16.01)	1704 (16.17)
	Married	8461 (71.98)	7896 (71.66)	7503 (71.21)
	Etc.(divorced, widowed)	1402 (11.93)	1358 (12.33)	1329 (12.61)
Education	Under elem.	2659 (22.62)	2510 (22.78)	2391 (22.70)
	High school	5709 (48.57)	5319 (48.28)	5064 (48.09)
	Over univ.	3387 (28.81)	3189 (28.94)	3076 (29.21)
Household income	Quantile 1	1712 (14.56)	1627 (14.77)	1580 (15.00)
	Quantile 2	2235 (19.01)	2178 (19.77)	2142 (20.33)
	Quantile 3	2655 (22.59)	2434 (22.09)	2337 (22.18)
	Quantile 4	2573 (21.89)	2386 (21.66)	2276 (21.60)
	Quantile 5	2580 (21.95)	2393 (21.72)	2201 (20.89)
Economic activity	No	4719 (40.14)	4357 (39.54)	4203 (39.89)
	Yes	7036 (59.86)	6661 (60.46)	6333 (60.11)
Insurance	Assistance	570 (4.85)	516 (4.68)	468 (4.44)
	NHI	1185 (95.15)	10,502 (95.32)	10,068 (95.56)
Disability	No	11,004 (93.61)	10,299 (93.47)	9819 (93.19)
	Yes	751 (6.39)	719 (6.53)	717 (6.81)
Total		11,755 (100.0)	11,018 (100.0)	10,536 (100.0)

*MCD* Multimorbidity, *SHS* Subjective Health Status, *MD* Medical Device

### Differences in independent, mediator, and dependent variables based on characteristics

Table 3 shows the mean and distribution of key variables according to the general characteristics based on the 2013 data. Women (showed higher morbidity from chronic diseases than men on average. Divorced/bereaved participants reported 2.32 diseases. The following groups also reported many chronic diseases: elementary school graduates or lower, income level in the first quantile, without economic activities, receiving medical benefits, and with disabilities. The same groups tended to perceive that their subjective health status was poor compared with the other groups, in terms of difference in subjective health condition according to general characteristics. Women tended to perceive poor health compared with men, whereas the others group for marital status perceived themselves as unhealthy.

**Table 3:** Difference in multimorbidity, assistive devices, self-rated health by explanatory variables

Variables		2011–2013			
		Multimorbidity^a^	Assistive devices^b^		Subjective Health Status^c^
			No	Yes	
Sex	Female	1.59 (1.27)	5443 (94.07)	343 (5.93)	2.77 (0.82)
	Male	1.27 (1.23)	4448 (93.64)	302 (6.36)	2.59 (0.81)
Marital status	Single	0.42 (0.77)	1596 (93.66)	108 (6.34)	2.34 (0.75)
	Married	1.52 (1.24)	7033 (93.74)	470 (6.26)	2.70 (0.79)
	Etc.(divorced, widowed)	2.32 (1.04)	1262 (94.96)	67 (5.04)	3.03 (0.89)
Education	Under elem.	2.43 (0.92)	2262 (94.60)	129 (5.40)	3.09 (0.86)
	High school	1.35 (1.24)	4761 (94.02)	303 (5.98)	2.62 (0.78)
	Over univ.	0.83 (1.05)	2864 (93.11)	212 (6.89)	2.48 (0.73)
Household income	Quantile 1	2.25 (1.10)	1486 (94.05)	94 (5.95)	3.05 (0.90)
	Quantile 2	1.66 (1.27)	2025 (94.54)	117 (5.46)	2.81 (0.82)
	Quantile 3	1.29 (1.24)	2196 (93.97)	141 (6.03)	2.65 (0.79)
	Quantile 4	1.14 (1.18)	2138 (93.94)	138 (6.06)	2.56 (0.76)
	Quantile 5	1.13 (1.16)	2046 (92.96)	155 (7.04)	2.47 (0.75)
Economic activity	No	1.77 (1.27)	3917 (93.20)	286 (6.80)	2.81 (0.90)
	Yes	1.23 (1.21)	5974 (94.33)	359 (5.67)	2.60 (0.76)
Insurance	Assistance	2.41 (1.03)	441 (94.23)	27 (5.77)	3.27 (0.96)
	NHI	1.40 (1.25)	9450 (93.86)	618 (6.14)	2.66 (0.80)
Disability	No	1.38 (1.25)	9230 (94.00)	589 (6.00)	2.65 (0.80)
	Yes	2.34 (1.01)	661 (92.19)	56 (7.81)	3.16 (0.93)

Note: ^a^Mean (SD), ^b^Frequency (ratio), ^c^Mean (SD)

### Logit and panel linear regression

Logit and panel regression analyses were conducted in three stages, with the results given in Table 4.

**Table 4:** Panel linear regression model for examining the mediated effect

		Step 1^a)^	Step 2^b)^	Step 3^b)^
		MD	SHS	SHS
Sex	Male	1.01	−0.12***	−0.12***
Age		1.00	0.01***	0.01*
Marital status (single)	Married	0.85	0.11***	0.11***
	Etc	0.64*	0.10***	0.10***
Education (under elem.)	High school	1.50***	−0.14***	− 0.15***
	Over univ.	1.71***	−0.15***	− 0.16***
Household income (quantile 1)	Quantile 2	1.07	−0.03	−0.03
	Quantile 3	1.10	−0.07***	−0.07***
	Quantile 4	1.14	−0.09***	−0.09***
	Quantile 5	1.45**	−1.15***	−0.16***
Economic activity	Yes	0.79**	−0.04**	−0.04**
Insurance type	NHI	1.10	−0.27***	−0.28***
Disability	Yes	1.65***	0.30***	0.29***
MCD		1.25***	0.15***	0.15***
MD	Yes			0.09***

Note: ^a)^ OR, ^b)^ Adjusted β, ^3)^ *p < .05, **p < .01, ***p < .001;

*MCD* Multimorbidity, *SHS* Subjective Health Status, *MD* Medical Device

First, the panel data were analyzed using logit regression regarding the impact of multimorbidity on using medical and assistive devices; the odds of using medical and assistive devices were lower for those who were divorced, living separated from their spouse, or bereaved of their spouse (OR = 0.64).

Meanwhile, the odds were higher among participants who were high school and college graduates or higher (OR = 1.50, OR = 1.71), had high income (OR = 1.45), and had disabilities (OR = 1.65). Use of medical and assistive devices was at least 1.25 times higher among those with chronic diseases. Participants engaged in economic activities (OR = 0.79) tended to use devices less.

Second, a panel regression analysis was conducted to identify the impact of multimorbidity on subjective health satisfaction. The minus (−) beta value in the panel regression analysis indicated a positive impact on subjective health status. As shown in Table 1, a higher subjective health satisfaction score indicated lower health satisfaction. For instance, if the male sex has a positive effect on subjective satisfaction (b = − 0.12), then it means that men have a higher subjective satisfaction compared with women. The results also showed that using medical and assistive devices had a positive impact on satisfaction with subjective health among high school and college graduates (b = − 0.14, − 0.15), income groups of all quantiles other than the second quantile (b = − 0.07, − 00.09, − 1.15), economically active population (b = − 0.04), and users of insurance (b = − 0.27). In contrast, using medical and assistive devices had a negative impact regardless of marital status (b = 0.11, 0.10), and resulted in lower satisfaction with subjective health when the participants had disabilities (b = 0.30). In particular, using medical and assistive devices had a negative impact on satisfaction with subjective health for those with chronic diseases (b = 0.15).

Third, a panel regression analysis was conducted to identify the factors influencing satisfaction with subjective health by setting multimorbidity and the use of medical and assistive devices as independent variables. The results showed that higher multimorbidity (b = 0.15) and the use of medical and assistive devices (b = 0.09) had a negative impact on satisfaction with subjective health. Among the sociodemographic variables, satisfaction with subjective health was high among men (b = − 0.12), high school and college graduates or higher (b = − 0.15, − 0.16), income groups of all quantiles other than the second quantile (b = − 0.07, 0.09, − 0.16), economically active population (b = − 0.04), and users of insurance (b = − 0.28). Satisfaction with subjective health was low among women, regardless of marital status (b = 0.11, 0.10), and those with disabilities (b = 0.29).

### Sobel test

Table 5 shows the results of the Sobel test verifying the statistical significance of the mediating effect of medical and assistive devices between multimorbidity and subjective health status. The test results showed that the verification value was 4.04 (*p* < 0.001), thereby proving that the partial mediating effect of medical and assistive devices is statistically significant.

**Table 5:** Path analysis using the Sobel test

Path	Beta	SD	Test statistics
MCD ➔ MD	0.223	0.036	• 4.04^a^
MD ➔ SHS	0.101	0.019	

*MCD* Multimorbidity, *SHS* Subjective Health Status, *MD* Medical Device

^a^Zab〉 ± 1.96

## Discussion

This study positively analyzed the impact of multimorbidity on the subjective health status of patients using assistive devices, and as a result, the hypotheses were verified as follows:

First, the hypothesis that multimorbidity will have a negative impact on subjective health status was confirmed. Multimorbidity had a negative impact on subjective health status (affecting deterioration) [48, 49]. Thus, people with multimorbidity are not psychologically and physically satisfied with their health status.

Second, the hypothesis that multimorbidity will have an impact on using medical and assistive devices was supported. Previous studies have proved that the need for assistive devices increases in persons with multiple diseases, and the scope of assistive devices should be expanded to guarantee satisfactory everyday activities in those with incomplete functions due to chronic diseases [6, 7]. At present, people with higher educational backgrounds, higher income level, and those who engaged in more economic activities have been observed to have more experience in using assistive devices, pointing to a potential accessibility issue.

Third, the hypothesis that using assistive devices will have an impact on subjective health satisfaction was also confirmed. In previous studies, their positive impact on satisfaction with subjective health [11, 12, 14] was dismissed, whereas their negative impact on subjective health status [16, 17] was accepted. We believe the socio-cultural prejudice against using assistive devices among Koreans negatively affects the subjective health status. Further, it is difficult for the use of individual assistive devices to have a positive impact on subjective health status because most assistive devices covered by the Korean national health insurance are items used by people in poorer physical health, such as mobile toilets, bath chairs, mobile beds, and mobile tubs. Thus, it would be necessary to expand the scope of assistive devices to include medical devices, such as dental implants, medical devices for prosthetic human implantation, and medical lasers so as to increase the subjective satisfaction of those who use assistive devices.

Fourth, the hypothesis that using assistive devices will have a mediating effect between multimorbidity and subjective health status was verified: using assistive devices had a negative (−) impact on subjective health. Such a result goes against previous findings of a positive impact on satisfaction with using assistive devices [41, 42].

## Conclusion

The mediating effect of using assistive devices was verified, but the direction of the effect turned out to be deteriorated subjective health. In other words, in the impact of multimorbidity on subjective health, assistive device use had a negative impact (−) on subjective health. This is contrary to previous studies [41, 42] claiming a positive impact on satisfaction. Meanwhile, this result is consistent with previous findings [27, 28, 49, 50] that more chronic diseases in participants had a negative impact on subjective health. The following implications can be made based on the aforementioned conclusion: using assistive devices has a negative mediating effect on satisfaction with the subjective health of patients with chronic diseases.

First, based on the research finding that chronic disease had a negative impact on satisfaction with subjective health, there must be a change in perception regarding the basic direction of the national health promotion policy: toward managing chronic diseases for each life cycle. In particular, efforts must be made to increase satisfaction with subjective health by managing chronic disease from adolescence, rather than implementing follow-up management of chronic disease for older adults. Consequently, a high quality of life can be ensured, by increasing satisfaction with subjective health from reduced chronic diseases, and reducing medical expenses for the elderly population.

Second, medical devices are currently limited to assistive devices used to help the daily living and physical activities of beneficiaries of the national long-term care insurance. To provide for all citizens, it is necessary to expand the scope of medical devices beyond the concept of assistive devices to include such items as imaging and ultrasonic wave devices. The scope may even be further expanded to provide health insurance benefits for other items, such as wearable technology, transplant techniques, decision-making data, and medically relevant Internet of Things. Such a shift can ensure that assistive devices, including medical appliances, can have a positive impact on satisfaction with subjective health for patients of all ages that have chronic diseases [51].

Third, assistive devices in Korea mostly follow the standard set for the beneficiaries of national long-term care insurance, limiting usage. The threshold amount provided to purchase or rent assistive devices is KRW 1.6 million per user annually, which includes deductibles and the health insurance burden charge. Considering the relatively expensive assistive devices, such as wheelchairs and automatic beds, the limited amount may keep beneficiaries from accessing assistive devices. Kim, Nam [43] stated that when beneficiaries select assistive devices based on price, they fail to choose assistive devices that are needed because of the barrier of the annual limit; this also seems to affect satisfaction. Therefore, it is necessary to expand the concept of assistive devices as well as the scope of application to all users, and medical devices including assistive ones must be included in health insurance benefits.

Fourth, the variables related to medical devices used in the KHP are limited to assistive devices; this aspect requires expansion and addition of medical devices. It is necessary to add new medical devices that enable active movement and a normal life to the panel data variables.

Fifth, the fact that using assistive devices has a negative impact on subjective health status in Korea seems to be related with the country’s socio-cultural norms. Using assistive devices can have a labeling effect, and therefore, users may not be emotionally satisfied with using them even if these devices give them physical convenience [17]. Therefore, the public’s views on the use of assistive devices should be changed in Korean society, and the coverage of the National Health Insurance for assistive devices should be expanded so that assistive devices can be more widely used.

In sum, narrow scope for choice of assistive devices, and limited scope of health insurance benefits must change to ultimately lead to a positive mediating effect on using medical devices and on satisfaction with the subjective health of users with chronic diseases. The system must be one that embraces all ages.

Meanwhile, this study had limitations in undertaking in-depth analysis as the demographic characteristics in the medical panels in Korea varied, such as those in persons with disabilities or the elderly population, who usually have experience in using assistive devices. Therefore, further research should be conducted to verify the effects of assistive devices for specific groups of users.

In verifying the mediating effect of using assistive devices on satisfaction with subjective health of persons with chronic diseases, this study has significance in attempting a new change in perception on the medical devices market, which is expected to show constant growth along with the development of the Fourth Industry. In particular, there is more significance in verifying the mediating effect of using medical devices through panel data. The narrow scope of medical devices and limited range of participants and health insurance coverage restrict the possibility of generalizing the research findings. Therefore, follow-up research is anticipated to verify the mediating effect of medical devices using panel data that comprehensively includes all medical devices. In particular, future studies should investigate the mediating effects of assistive devices in the relationship between subjective satisfaction and such indices as the Cumulative Illness Rating Scale, Charlson Comorbidity Index, and Functional Comorbidity Index.
